# Ferulic Acid Treatment Maintains the Quality of Fresh-Cut Taro (*Colocasia esculenta*) During Cold Storage

**DOI:** 10.3389/fnut.2022.884844

**Published:** 2022-05-24

**Authors:** Bin Wang, Yongyan Huang, Zhenming Zhang, Yanhui Xiao, Jing Xie

**Affiliations:** Shaoguan Aromatic Plant Engineering Research Center, Henry Fok College of Biology and Agriculture, Shaoguan University, Shaoguan, China

**Keywords:** fresh-cut taro, cold storage, surface discoloration, ferulic acid, aroma quality

## Abstract

Taro (*Colocasia esculenta*) is a major root crop or vegetable in the world, and the corm is a good source of many nutrients including starch, vitamins, and minerals. Taro corms are processed into various forms before consumption, which makes them perishable, reduces the shelf life, and increases postharvest losses. The surface browning of fresh-cut taros is one of the major factors that limits storage life and affects consumer acceptance. In this study, the effects of ferulic acid (FA) as an effective agent in the prevention of quality deterioration were investigated. Fresh-cut taros were immersed in distilled water and different concentrations of FA (1, 2, 5, 10, and 20 mM) solutions for 30 min, air-dried at 25°C for 30 min, and then stored at 5°C for 12 days to investigate the effects of FA on browning. Among the FA concentrations tested, 10 mM resulted in significantly higher L^*^ values, lower a^*^ and b^*^, and browning index values. FA treatment (10 mM) also induced *de novo* biosynthesis of two volatile compounds, including non-anal and octanoic acid ethyl ester in fresh-cut taros following extended cold storage. The results suggest that FA treatment maintains the quality of fresh-cut taros under cold conditions. FA treatment enhanced PAL activity and gene expression but reduced total phenolic content and the expression of six *C4H, 4CL*, and *CHS* genes, suggesting that FA treatment reduced phenolic biosynthesis. FA treatment reduced PPO activity and gene expression and decreased soluble quinone content, suggesting that FA treatment suppressed the phenolic oxidation. FA treatment enhanced the activity and gene expression of CAT and POD, reduced those of LOX, and decreased MDA and H_2_O_2_ levels, suggesting that FA treatment activated the antioxidant defense system and thereby reduced oxidative damage. These findings demonstrated that FA treatment could serve as an effective approach to retard the browning of fresh-cut taros and provided a basis for the feasible application of FA in the preservation of fresh-cut foods.

## Introduction

In recent years, consumer demands for the consumption of fresh-cut fruits and vegetables have dramatically expanded because of the convenience, freshness, and lack of pollution of fresh-cut products (1, 2). However, cutting and peeling operations cause mechanical damage to fresh products and result in the release of cellular contents, which brings about a series of undesirable consequences such as the growth of harmful microbes, cut-surface browning, stale taste, and short shelf life (3). Among the undesirable changes, the discoloration of cut surfaces is the major factor affecting storage life and consumers' acceptance (4, 5). Therefore, it is necessary to develop more effective postharvest techniques that can not only maintain the quality but also do not affect the safety of fresh-cut foods.

The postharvest methods used to prevent the discoloration of fresh-cut products mainly include physical and chemical treatments (6). The inhibitory efficacy of physical treatments, such as washing, UV sanitizing, edible coating, heat treatment, modified atmosphere packaging, and cold storage, has been extensively investigated (7–12). Cold storage is the most effective among physical method that maintains the storage quality of fresh-cut foods. However, the browning of fresh food is generally caused by enzymatic reactions, and the browning-related enzymes in fresh-cut products are still active under low-temperature conditions (13, 14). Therefore, the combination of cold storage with chemical additives may be a more effective strategy (15–17). Among the chemicals studied, sulfite- and chlorine-containing additives are the most effective, and as a result, they have been widely used in the fresh-cut industry in the past years (18, 19). However, the use of these chemicals may lead to concerns from consumers, as such compounds have potential hazards to human health. Therefore, more safe browning inhibitors are worth developing.

Taro (*Colocasia esculenta* L.) belongs to the *Araceae* family (20). It is similar to other root vegetables, such as potatoes, Chinese water chestnut (CWC), burdocks, yams, and lotus roots, with the root being the main edible organ (21). In China, the peels of taro are removed before cooking (22). However, fresh-cut taros get spoiled more easily than the intact taros, because peeling and cutting can result in the loss of compartmentation of enzymes and substrates, which leads to browning (22). Therefore, the peeled taros brown easily during the period of shelf life and consumption (23). The application of two kinds of hydrosols from citronella plants and rose petals to fresh-cut taros was effective in reducing the browning under cold conditions (24). Further analysis indicated the presence of carboxylic acids in abundance in two hydrosols. Carboxylic acids belong to organic acids and are widely distributed in the plant kingdom (25). It has been reported that several carboxylic acids or their derivatives, such as cinnamic acid, phytic acid, citric acid, coumaric, caffeic, and chlorogenic acid, have potential effects on the browning inhibition of fresh-cut products (6, 26, 27). The application of phytic acid to fresh-cut purple sweet potatoes markedly inhibited the browning caused by polyphenol oxidases (PPO) (28). Cinnamic acid was an effective browning inhibitor in preventing the browning development of fresh-cut taros during cold storage (22). In general, carboxylic acids can interact with metal ions, which enable them to scavenge free oxidative radicals (29).

Ferulic acid (FA), 4-hydroxy-3-methoxycinnamic acid, is one of the carboxylic acids naturally existing in many plants (29, 30). FA is one of the most abundant phenolic acids in plants, whose contents vary from 5 g kg^−1^ in wheat bran to 50 g kg^−1^ in corn kernels (30). Song et al. (31) first reported that the application of 10 mM FA to fresh-cut CWC effectively prevented the yellow color development and inhibited phenylalanine ammonia-lyase (PAL), peroxidase (POD), and PPO activities. However, studies show that CWC yellowing is different from enzymatic browning (31, 32). Therefore, more investigations are needed to determine whether FA could reduce the browning development of fresh-cut taros, as the browning type of fresh-cut taro may be enzymatic (21, 23, 33). Furthermore, since FA is a precursor in the production of many aromatic compounds whether and how FA treatment influences the aroma quality of fresh-cut taros under cold storage conditions remains unclear.

This study investigated the effects of FA treatment on the browning reduction of fresh-cut taros. In addition, the effects of FA treatment on the contents of total phenolic compound (TPC), soluble quinone (SQ), hydrogen peroxide (H_2_O_2_), and malondialdehyde (MDA), as well as the activities and gene expression of PAL, PPO, POD, catalase (CAT), and lipoxygenase (LOX) were analyzed. Moreover, the effects of FA treatment on the aroma quality of fresh-cut taros following a long-term cold storage were evaluated. The results would lay the foundation for the practical application of FA in the fresh-cut industry. The investigations on FA effects on quality maintenance would reduce the postharvest losses of fresh-cut products and provide alternatives for the preservation of fresh-cut products.

## Materials and Methods

### Materials Utilized, Treatments, and Sampling Procedures

Taro (cv. binglang) roots were purchased from a local market in Shaoguan City, Guangdong province, China. We first booked taro materials the day before the investigation and made the demands including maturity and weight. Taro corms at commodity maturity were used for investigations (22). The corms were then transported to the laboratory of the Shaoguan Aromatic Plant Engineering Research Center, Shaoguan University, within 48 h of harvest. Taro corms were selected with uniform shape and size, and a lack of physical injury or disease signs. The selected corms were washed with tap water before being peeled and cut into 1-cm thick slices (6–8 pieces for each root) using a stainless steel knife. Then, the slices were soaked in the distilled water (DW) and different concentrations of FA (1, 2, 5, 10, and 20 mM) solution for 30 min and air-dried at room temperature (25°C) for 30 min. Finally, the treated taro slices were packed with plastic film (0.02-mm thick polyethylene) and stored at 5°C for 12 days.

Each treatment contained 60 slices with three replicates. Taros from the DW and each treatment were randomly sampled for further investigations. Three slices from the same treatment were chopped and pooled using a stainless steel knife, and then the mixed samples were ground to powder in liquid nitrogen and stored at −80°C.

### Color Measurement

The color change of the taro slice surface was quantified using a digital Chroma Meter (CR-400, Konica Minolta, Japan), which presents the L^*^, a^*^, and b^*^ values using the CIELAB scale. The chroma meter was calibrated using a standard white tile (Y = 84.4, x = 0.3205, and y = 0.3377) before measurement. The L^*^, a^*^, and b^*^ values of each test were converted into browning index (BI) according to the following formulas: BI = [100(*x* – 0.31)] / 0.17, *x* = (a^*^ + 1.75L^*^) / (5.645L^*^ + a^*^ – 3.012b^*^) (22). After color measurement, pictures were taken at each sampling point.

### Determination of Total Phenolic Compound (TPC) and Soluble Quinones (SQ) Contents

The TPC of fresh-cut taros was evaluated by using the method described by Liu et al. (34). The TPC content of taro slices was measured using the Folin–Ciocalteu method with a UV-Spectrophotometer (UV-6100, SHjingmi, China) at 765 nm. Gallic acid was employed as a calibration standard, and the results were expressed as gallic acid equivalents (GAE) per kilogram of fresh weight (mg GAE kg^−1^ FW) (35).

Soluble quinone concentrations were determined according to the method described by Ali et al. (36). The 5.0 g of taro samples from three slices were ground using 10 ml of methanol at room temperature (25°C) and centrifuged at 12,000 × *g* for 20 min. The absorbance of each supernatant at 437 nm was measured, and the SQ concentrations were expressed as OD437 per gram of fresh weight (OD437 g^−1^ FW).

### Measurement of MDA and H_2_O_2_ Concentrations in Fresh-Cut Taros

The MDA and H_2_O_2_ contents were estimated using commercial kits (D799761 and D799773, Sangong Biotech, China) according to the manufacturer's instructions. The MDA and H_2_O_2_ contents were measured using 0.1 g of taro powders from three different slices.

The MDA content = [6.45 × (A532–A600) – 0.56 × A450] × Vt / (Vs × m) (37), where Vs represents the extract volume required for determination and Vt represents the total volume of sample extract. The concentration was expressed as nmol per kilogram of fresh weight (nmol kg^−1^ FW). For the measurement of H_2_O_2_ content, the absorbance of the solution was recorded at 415 nm, and the content was reported as μmol per kilogram of fresh weight (μmol kg^−1^ FW).

### Extraction Procedure for Enzymatic Assay

The 1.0 g of composite pulverized samples was extracted in 5 ml of 0.5 mM phosphate buffer (pH 6.8) containing 2% of polyvinylpyrrolidone. The extractions were centrifuged at 12,000 × *g* for 10 min at 4°C. The supernatants were collected and regarded as crude enzyme extractions.

The activities of CAT, POD, PPO, PAL, and LOX in fresh-cut taro samples were estimated with the assay of Xiao et al. (22), and activities of all above enzymes were expressed as unit per kilogram of fresh weight (U kg^−1^ FW). The CAT activity was measured by monitoring the decomposition rate of H_2_O_2_ at 240 nm in a reaction with 10 mM H_2_O_2_ using a UV-visible spectrophotometer. The POD activity was determined by measuring guaiacol oxidation at 470 nm in reaction with 20 mM concentrated guaiacol (36). The PPO activity was assayed by monitoring the decrease of 4-methylcatechol at 398 nm (24). For the PAL activity assay, 0.2 ml of the supernatants, 0.5 ml of 5 mM dithiothreitol, 0.5 ml of 0.02 M l-phenylalanine, and 1.8 ml of 0.1 mM sodium borate (pH 8.8) were reacted at room temperature (25°C) for 2 h, and the absorbance was noted at 290 nm. For the measurement of LOX activity, a reaction mixture was made, which contained 0.1 ml of the supernatants, 0.2 ml of 5 mM linoleic acid, and 2.7 ml of 0.5 M borax–borate buffer (pH 7.0). LOX activity was determined on the basis of the formation of conjugated dienes at 234 nm (4).

### Gene Expression Analysis

Total RNA from taro samples was extracted with the RNAprep Pure Plant Plus Kit (DP441, Tiangen, China), and one-strand cDNA was synthesized with an iScript cDNA Synthesis Kit (11123ES60, Yeasen, China) according to the manufacturer's instructions (22). A specific primer for each gene was designed using a primer designing tool at the National Center for Biotechnology Information (NCBI) website, and primer details were listed in Supplementary Table 1. Quantitative real-time PCR (qRT-PCR) was performed using an iCyeler iQTM/Clooo system (Bio-Rad, USA), and a Hieff® qPCR SYBR Green Master Mix (No Rox) kit was used (11201ES03, Yeasen). Relative expression of the target gene was normalized to taro Actin7 as the internal control. mRNA sequences of candidate genes for qRT-PCR analysis were isolated from the taro genome (https://www.ncbi.nlm.nih.gov/genome/12429) using BLAST searches of the published sequences.

### Detection of Volatile Compounds in Fresh-Cut Taros

A gas chromatography (GC) system (Agilent 7890B, USA) coupled with mass spectrometry (MS; Agilent 5977B, USA) equipped with an automated headspace sampler (CTC Analytics AG, Switzerland) was used for the analysis of volatile components in DW- and FA-treated taros (38, 39). Fresh taro samples on days 0 and 12 were used for investigations.

The headspace and GC operating conditions refer to the descriptions of Zhang et al. (40), with minor modifications. Approximately, 2.0 g of taro samples were weighed in a GC-MS vial (20 ml). The GC-MS analysis program was started, and the vials were placed in a thermostatic stirrer at 100°C for 5 min. The headspace operating conditions were as follows: equilibration temperature, 160°C; equilibration time, 60 min; pressing time, 0.5 min; extracting time, 0.2 min; and injecting time, 0.5 min. The injection port temperature was maintained at 230°C, and the split ratio was 10:1.

Helium (99.99% purity) was used as the carrier gas at a flow rate of 1.0 ml/min. Separation of compounds was achieved using a quartz capillary column (HP-5MS UI 30 m × 0.25 mm with 0.25 μm film thickness). The initial oven temperature was set at 50°C for 1 min and gradually increased to 230°C at a rate of 5°C/min with a holding time of 1 min. The analysis time was 31 min. The temperatures of the ion source temperature and vacuum chamber were maintained at 200 and 150°C, respectively. The ionization energy was 70 eV, and the emission current was 35 mA. The mass range was 33–520 m/z, and the scan rate was 11.7 scans per second.

Compound identifications were done by matching the MS spectra and comparing the retention time with that of a corresponding reference standard after all compounds were successfully separated.

### Statistical Analysis

All investigations were performed in triplicate, and the results were presented as means ± standard deviation (SD, *n* = 3). Mean and SD values from their independently replicated sets were calculated using the Excel software (version 2016). Statistical differences between treatments were analyzed by one-way ANOVA using the SPSS software package (version 21) at the *p* ≤ 0.05 level. The differences were presented with different letters above the bars in the figures.

## Results

### Effects of Different Concentrations of FA Treatments on Color Value of Fresh-Cut Taro During Cold Storage

**Figure 1:**
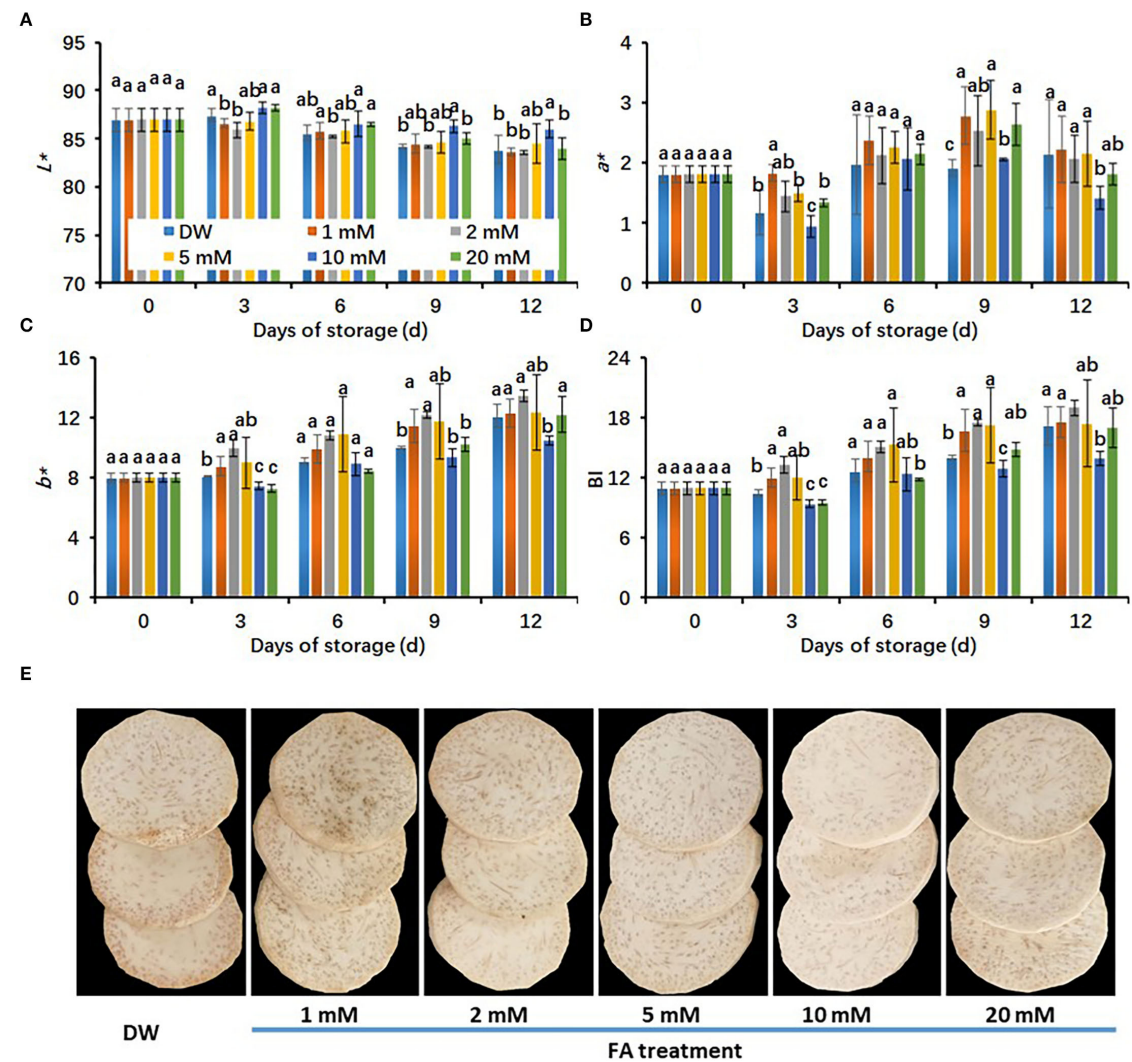
Effects of different concentrations of FA treatments on the browning development of fresh-cut taro during storage at 5°C. **(A)** L* value; **(B)** a* value; **(C)** b* value; **(D)** browning index (BI), **(E)** the representative pictures of fresh-cut taros, which were taken at 12 days of cold storage at 5°C. Each value is presented as means ± standard deviation (SD, *n* = 3). Statistical differences (*p* ≤ 0.05) between treatments at the same time point are analyzed and indicated using different letters above the bars.

In order to understand the effects of FA treatments on the color value of fresh-cut taro under cold storage conditions, we measured the L^*^ value because it is an indicator of color brightness. In all treatments, L^*^ values decreased with storage time (Figure 1A). The L^*^ values of taro slices did not show significant differences among the control (DW-treated group), 1, 2, 5, and 20 mM FA-treated groups. However, the 10 mM FA-treated group showed the highest L^*^ values, which were significantly higher than those of the control during cold storage (Figure 1A). On the whole, increased trends in a^*^ and b^*^ values and browning index (BI) of fresh-cut taros were observed throughout the cold storage period (Figures 1B–D). In particular, 10 mM FA treatment was the most effective at restricting the increase of a^*^ and b^*^ values and BI in taro slices during the cold storage period (Figures 1B–D). On day 6, the browning symptoms started to appear in DW-treated taro slices, revealing the effectiveness of cold storage on the browning suppression of fresh-cut taros. However, the browning symptoms in the control were obviously aggravated during the late storage period. The effects of FA treatments on the browning inhibition of taro slices on day 12 are presented in Figure 1E.

**Figure 2:**
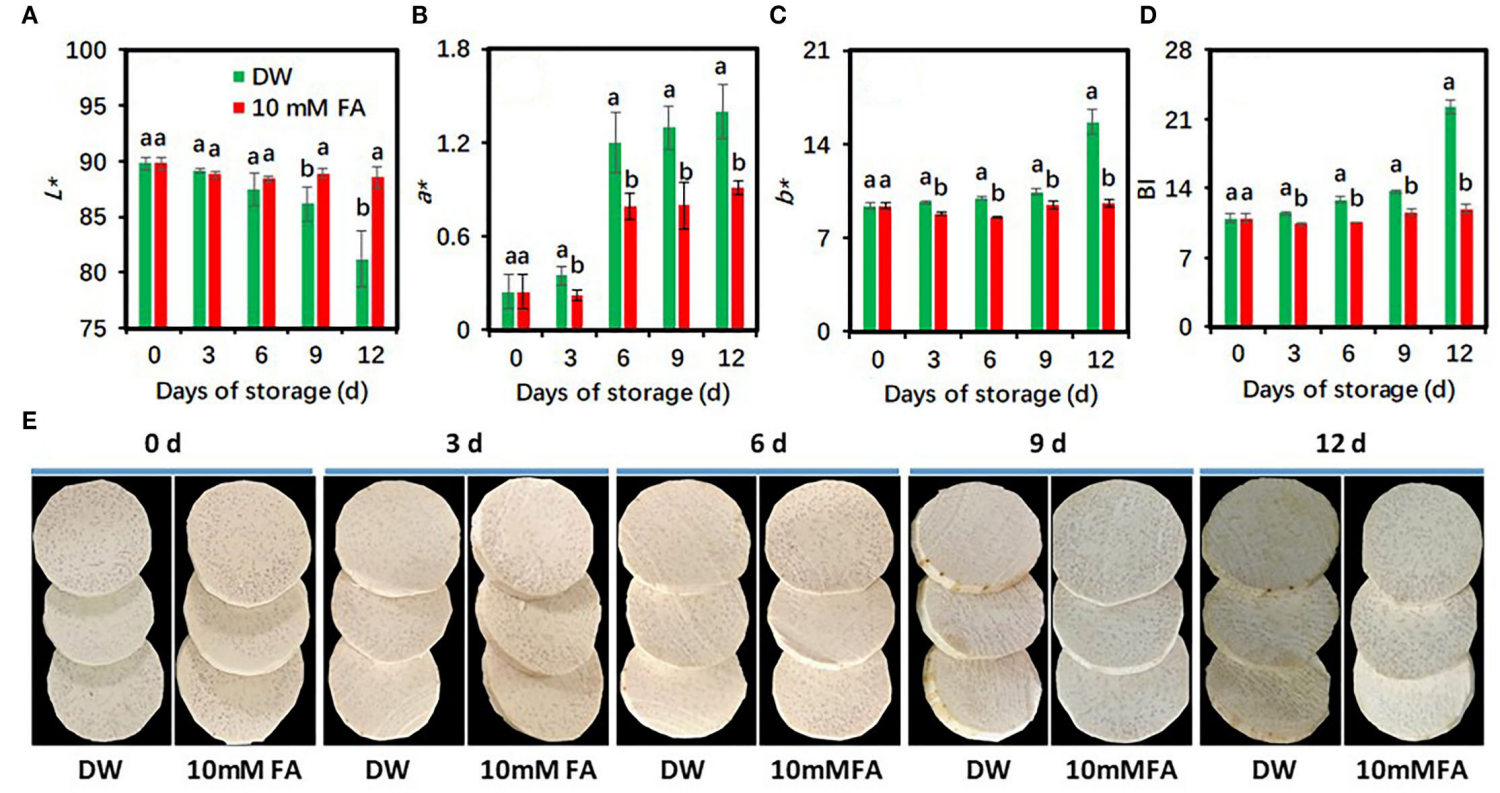
Effects of 10 mM FA treatment on the browning development of fresh-cut taro during storage at 5°C. **(A)** L* value; **(B)** a* value; **(C)** b* value; **(D)** browning index (BI), **(E)** the representative pictures of fresh-cut taros, which were taken at each sampling point. Each value is presented as means ± standard deviation (SD, *n* = 3). Statistical differences (*p* ≤ 0.05) between the control and 10 mM FA treatment at the same time point are analyzed and indicated using different letters above the bars.

The concentration of FA 10 mM shows a very slight browning reaction in fresh-cut taros during the cold storage period. To confirm the effectiveness of browning inhibition of 10 mM FA treatment, we carried out a new investigation. The peeled, fresh-cut taros were immersed into DW and 10 mM FA solutions for 30 min, respectively. The storage conditions for this experiment were the same as for the previous experiment. Undoubtedly, the 10 mM FA treatment brought about higher L^*^ values, but lower a^*^ and b^*^ values and BI, resulting in a brighter appearance than the control during the entire storage period (Figure 2). Overall, our results suggested that 10 mM FA treatment could reduce the browning and maintain the visual quality of fresh-cut taro under cold storage conditions. Therefore, taro samples exposed to this treatment were used for further study.

### FA Treatment Regulates the Activity and Gene Expression of Enzymes in the Phenylpropanoid Pathway in Fresh-Cut Taros

**Figure 3:**
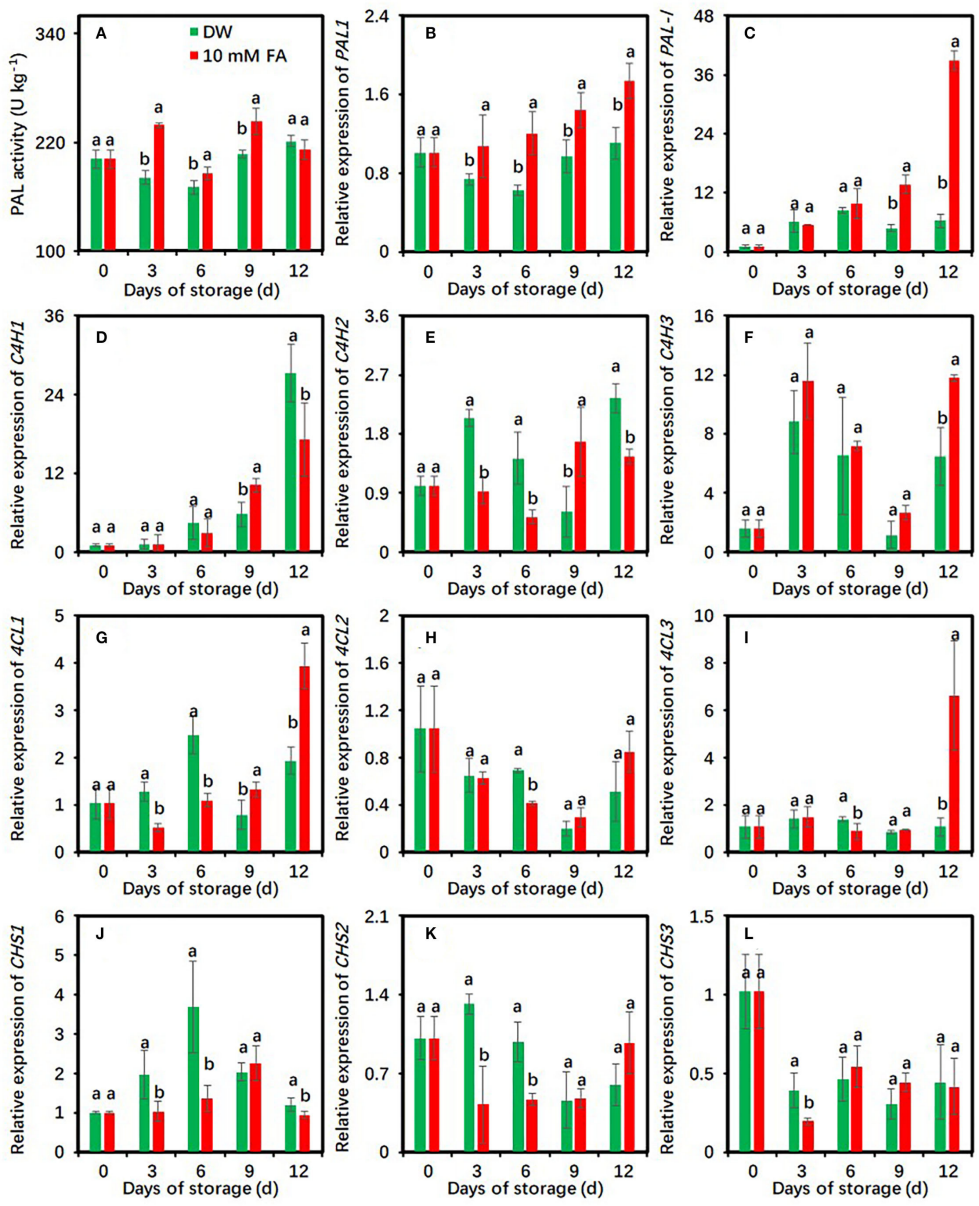
Effects of 10 mM FA treatment on the activity and gene expression of enzymes in the phenylpropanoid pathway in taro slices during storage at 5°C. **(A–C)** PAL activity and two *PAL* gene expression; **(D–F)** three *C4H* gene expression; **(G–I)** three *4CL* gene expression; **(J–L)** three *CHS* gene expression. Each value is presented as means ± standard deviation (SD, *n* = 3). Statistical differences (*p* ≤ 0.05) between the control and 10 mM FA treatment at the same time point are analyzed and indicated using different letters above the bars.

PAL activities in the control and 10 mM FA-treated groups were first increased on days 12 and 3 as compared with the initial day of storage, respectively (Figure 3A). Compared with the control, FA treatment significantly enhanced PAL activities during cold storage at each time point except for day 12 (Figure 3A), indicating that FA treatment could induce PAL activities in fresh-cut taros. To verify the induction of FA treatment, the effects of FA treatment on the expression of two *PAL* genes, namely, *PAL1* and *PAL*-like genes, were also investigated. The expression levels of *PAL1* in FA treatment were significantly higher than those in control groups during the entire storage period (Figure 3B). Compared with the control, the expression levels of *PAL*-like gene were significantly upregulated in the last 6 days (Figure 3C).

The effects of FA treatment on the expression patterns of cinnamate-4-hydroxylase (C4H), 4-coumarate: CoA ligase (4CL), and chalcone synthase (CHS)-encoded genes were also studied. Compared with the control, FA treatment significantly repressed *C4H1* expression on day 12 and *C4H2* expressions during the whole storage period with an exception on day 9, respectively (Figures 3D,E). However, FA treatment did not significantly suppress the expressions of *C4H3* and *4CL3* during the entire storage. Their expression levels in the control were higher than those in the FA-treated group on day 12 (Figures 3F,I). The expressions of *4CL1* and *4CL2* were significantly repressed by FA treatment on day 3 and day 6 compared with the control (Figures 3G,H). For the three *CHS* genes, FA treatment significantly restrained *CHS1* expression during the entire storage, with an exception on day 9, *CHS2* expression on days 3 and 6, and *CHS3* expression on day 3, respectively (Figures 3J–L).

### Effects of FA Treatment on Oxidation Characteristics of Fresh-Cut Taro Slices

**Figure 4:**
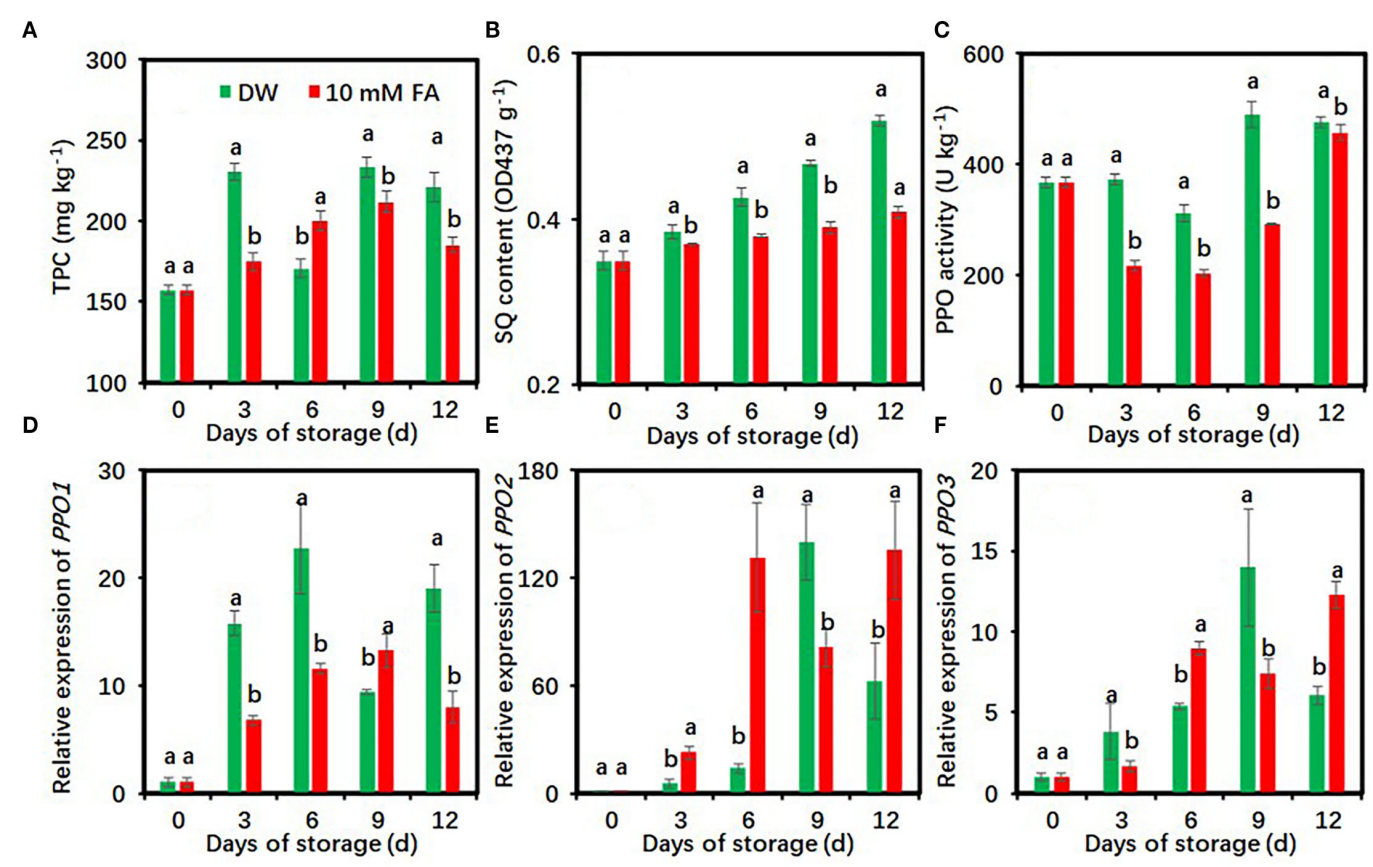
Effects of 10 mM FA treatment on total phenolic contents **(A)**, soluble quinone contents **(B)**, and the activity and gene expression of PPO **(C–F)** in taro slices during storage at 5°C. Each value is presented as means ± standard deviation (SD, *n* = 3). Statistical differences (*p* ≤ 0.05) between the control and 10 mM FA treatment at same time point are analyzed and indicated using different letters above the bars.

The contents of total phenolic compounds showed an upward trend during the whole storage period, while the 10 mM FA treatment significantly reduced total phenolic contents during cold storage except on day 6 (Figure 4A). SQ contents progressively increased along with storage days. Compared with the control, FA treatment significantly restricted the increase of SQ contents during the whole storage period (Figure 4B). FA treatment reduced SQ content by 3.98–21.44% compared with the control.

The PPO activity decreased in the first 6 days and then increased and was maintained at a high level during the late storage period (Figure 4C). Compared with the control, FA treatment significantly reduced PPO activity during the whole cold storage period (Figure 4C). During the 9th day of storage, the PPO activity of the control group was 1.68-fold higher than that of the FA-treated group. FA treatment downregulated the expression of three *PPO* genes at several time points (Figures 4D–F). The *PPO1* expression level of FA treatment on days 3, 6, and 12, the *PPO2* expression level on day 9, and the *PPO3* expression level on days 3 and 9 were significantly lower than those of the control, respectively (Figures 4D–F).

### Effects of FA Treatment on Antioxidant Activity of Fresh-Cut Taros

**Figure 5:**
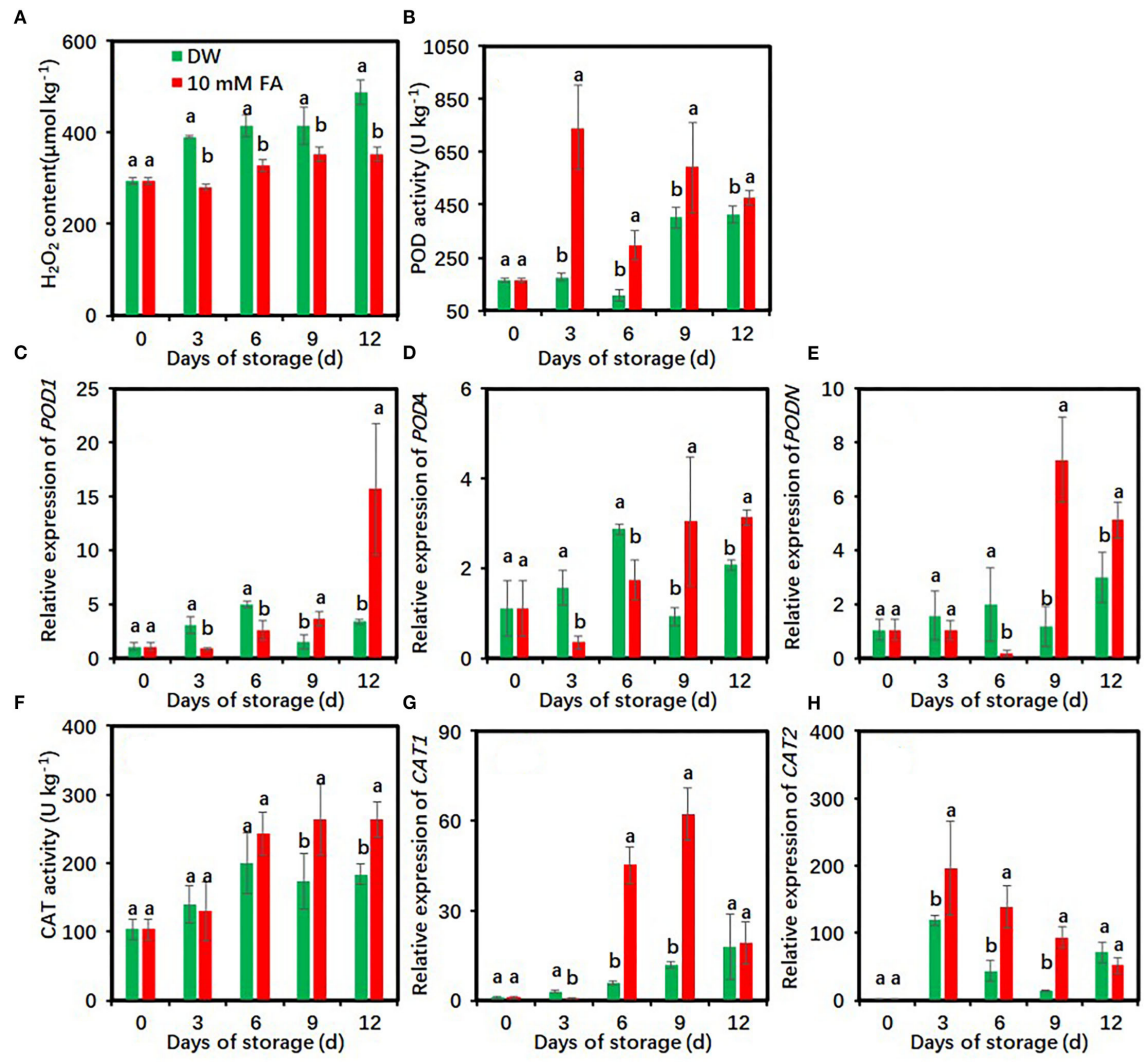
Effects of 10 mM FA treatment on hydrogen peroxide contents **(A)**, and the activity and gene expression of POD **(B–E)** and CAT **(F–H)** in taro slices during storage at 5°C. Each value is presented as means ± standard deviation (SD, *n* = 3). Statistical differences (*p* ≤ 0.05) between the control and 10 mM FA treatment at the same time point are analyzed and indicated using different letters above the bars.

Hydrogen peroxide content rapidly increased in the control but increased slowly in the FA-treated group within 12 days of measurements (Figure 5A). Compared with the control, FA treatment significantly reduced H_2_O_2_ content during the entire storage period.

Peroxidase activity of the control first increased on day 6, rose to a peak on day 9, and then decreased (Figure 5B). POD activity in the FA-treated group increased rapidly within the first 3 days and then maintained at high levels during the late storage. Compared with the control, FA treatment significantly enhanced POD activity during the whole cold storage period (Figure 5B). On day 3, the POD activity of the control was 4.20-fold higher than that of the FA treatment. The expression levels of three POD*-*encoded genes in the FA-treated group were significantly higher than those in the control within the last 6 days but lower during the first 6 days (Figures 5C–E).

The CAT activity rapidly increased during the first 6 days and was maintained at high levels during the remaining storage period in both the DW- and FA-treated groups (Figure 5F). Compared with the control, FA treatment significantly enhanced CAT activity on days 9 and 12 of cold storage (Figure 5F). The expression level of *CAT1* in the control was progressively increased in the entire storage, whereas the expression after FA treatment dramatically increased on days 6 and 9 and showed a low level on day 12 (Figure 5G). The *CAT2* expression level sharply increased to a peak on day 3 and declined subsequently (Figure 5H). Compared with the control, FA treatment significantly upregulated *CAT1* expression on days 6 and 9 and *CAT2* expression on days 3, 6, and 9, respectively (Figures 5G,H).

### FA Treatment Alleviates the Peroxidation of Membrane Lipids of Fresh-Cut Taro Slices

**Figure 6:**
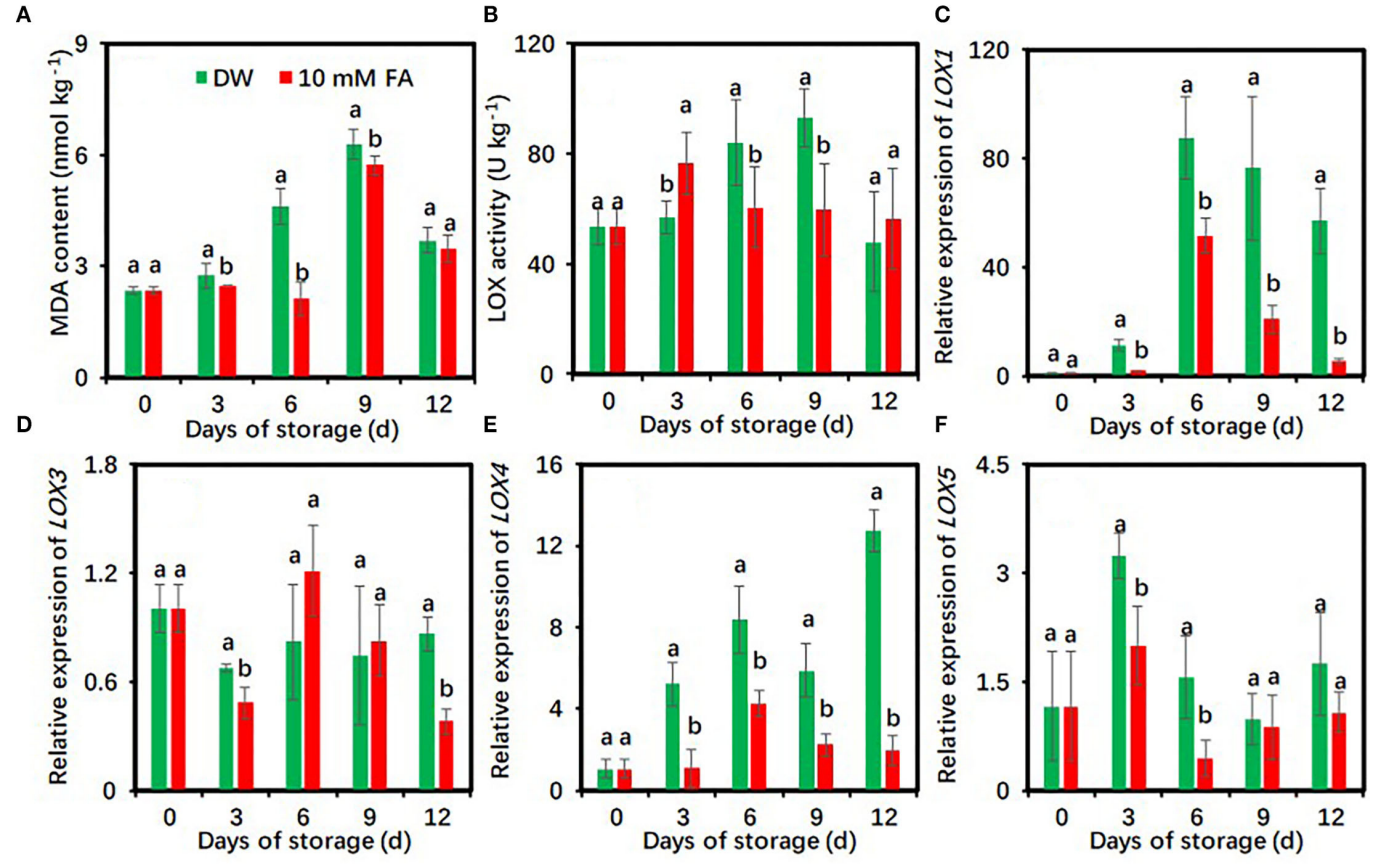
Effects of 10 mM FA treatment on malondialdehyde contents **(A)** and the activity and gene expression of LOX **(B–F)** in taro slices during storage at 5°C. Each value is presented as means ± standard deviation (SD, *n* = 3). Statistical differences (*p* ≤ 0.05) between the control and 10 mM FA treatment at the same time point are analyzed and indicated using different letters above the bars.

Malondialdehyde levels of fresh-cut taros in both groups showed peaks on day 9 (Figure 6A). FA treatment resulted in significantly lower MDA content relative to the control during cold storage except on day 12 (Figure 6A). The LOX activities of DW-treated taros steadily increased and showed a peak at day 9, whereas those of FA-treated taros changed much less (Figure 6B). Compared with the control, FA treatment significantly reduced LOX activity on days 6 and 9.

The effects of FA treatment on the expression of four *LOX* genes were also investigated. FA treatment significantly downregulated the expressions of *LOX1* and *LOX4* genes during the entire period of cold storage, *LOX3* expression on days 3 and 12, and *LOX5* expression on days 3 and 6 compared with the control (Figures 6C–F).

### Effects of FA Treatment on the Aromatic Characteristics of Fresh-Cut Taros

**Figure 7:**
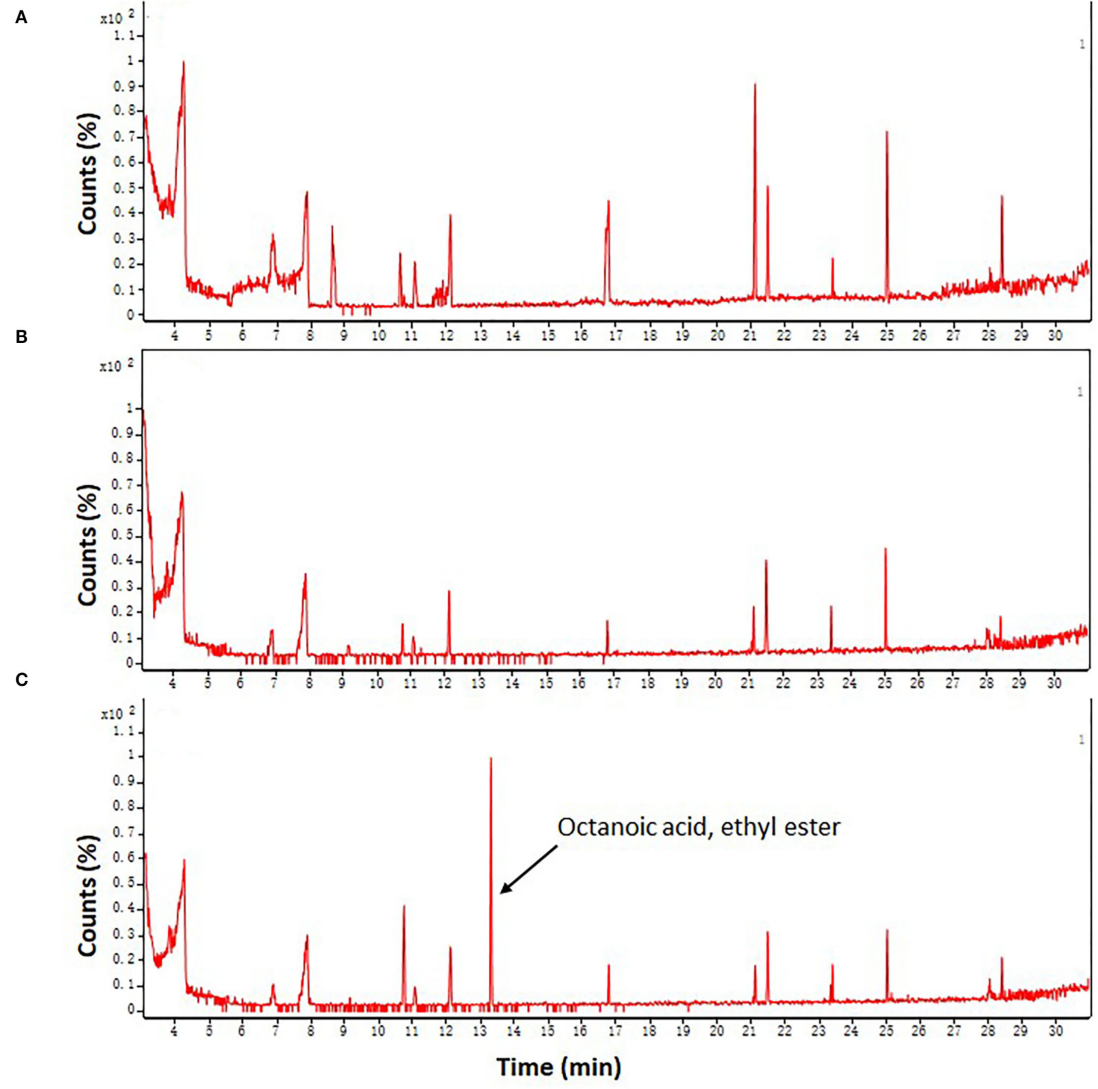
Plots of FID chromatogram of fresh-cut taros using a GC-MS system. **(A)** Fresh samples from 0 day of storage; **(B,C)** DW-treated and FA-treated samples from 12 days of storage at 5°C, respectively. Each sample was repeatedly analyzed three times. The representative plot shown were obtained from three replications.

Given that FA is an intermediate in the biosynthesis of various aromatic compounds in the phenylpropanoid pathway (41), therefore we further analyzed whether and how FA treatment influences the aroma quality of fresh-cut taros following long-term cold storage. Rare volatile compounds were identified in taros by using a GC-MS assay (Figure 7). Before cold storage, a total of six volatile components were successfully identified in fresh taros (Supplementary Table 2). However, only two kinds of volatile compounds were identified in DW-treated taro slices after 12 days of cold storage (Supplementary Table 2).

After 12 days of cold storage, five compounds were totally found in the FA-treated taro slices. Among these five compounds, three compounds, including cyclopentasiloxane decamethyl, 2,4-di-tert-butylphenol, and 18-methyl-non-adecane-1,2-dio trimethylsilyl ether, were also identified in fresh taros on the initial day of storage. The relative content of cyclopentasiloxane decamethyl was increased but the relative content of 2,4-di-tert-butylphenol was reduced after FA treatment compared with the control at day 12. The relative content of 18-methyl-non-adecane-1,2-dio trimethylsilyl ether did not change before and after FA treatment (Supplementary Table 2). Moreover, two novel compounds, namely, non-anal, and octanoic acid ethyl ester, with a distinctive aroma were discovered in FA-treated taros after 12 days of cold storage (Supplementary Table 2 and Figure 7). These two aromatic compounds were newly biosynthesized after FA treatment.

## Discussion

The surface color is a visual indicator of good quality as this cosmetic feature highly correlates with the freshness of fresh-cut products such as taro corm (21, 22). The parameters of L^*^ value, a^*^ value, b^*^ value, and BI are regarded as indicators of browning severity in fresh-cut taros (24). In this study, the efficiency of FA treatments with different concentrations (1, 2, 5, 10, and 20 mM) on the browning inhibition in fresh-cut taros was evaluated. The results indicated that 10 mM FA significantly reduced the browning reaction in fresh-cut taros (Figure 1), as reflected by the results that the decrease of L^*^ values, the increase of a^*^ and b^*^ values, and the BI of taro slices were significantly inhibited by 10 mM FA treatment (Figures 1, 2). These results suggest that the inhibition efficiency of FA treatment on taro browning depends on FA concentrations used, varying from 1 to 20 mM FA. It has been reported that 10 mM FA treatment can significantly reduce the discoloration of fresh-cut CWCs (31). The results demonstrated that 10 mM FA treatment was effective in reducing the browning of fresh-cut products.

The *de novo* biosynthesis of phenolic compounds induced by peeling and cutting can promote the browning development of fresh-cut products (31, 32). The phenylpropanoid pathway is one of the chief pathways responsible for phenolic biosynthesis in plants (41). PAL, C4H, 4CL, and CHS are the four key enzymes in the phenylpropanoid pathway (42). The activity of such enzymes in fruit and vegetables can be activated by fresh-cut operations (43). An increase in phenolic content was related to the aggravated browning in many fresh-cut products (43, 44). Moreover, FA is a key upstream intermediate of the phenylpropanoid pathway and is the precursor for the biosynthesis of many phenolics (41). To explore whether the external addition of FA could influence the biosynthesis of phenolic compounds in fresh-cut taros, the effects of FA treatment on total phenolic content, PAL activity, and the expressions of three main enzyme-encoded genes, including C4H, 4CL, and CHS, were investigated.

Phenylalanine ammonia lyase is an entry-point enzyme in phenolic biosynthesis through the phenylpropanoid pathway (41). The results currently available about the effects of exogenous FA treatment on PAL activity are contradictory. For example, Sato et al. reported that FA is ineffective against PAL activity in sweet potatoes (45). As a feedback regulator, FA inhibits PAL activity and gene expression in fresh-cut CWCs (31). In contrast, FA functions as an inducer of PAL activity in soybean roots (46). In this study, FA treatment enhanced PAL activity and upregulated the expressions of two *PAL* genes (Figures 3A–C), suggesting that FA treatment activated PAL activity in fresh-cut taros.

Cinnamic acid is the first product in the phenylpropanoid pathway (47). PAL directly catalyzes the non-oxidative deamination of l-phenylalanine and l-tyrosine to form cinnamic acid (48). These results suggest that FA treatment could increase endogenous cinnamic acid content in fresh-cut taros by inducing PAL activity. The application of cinnamic acid on fresh-cut taros reduces taro browning (22), suggesting that FA treatment reduces taro browning partly through inducing cinnamic acid biosynthesis.

In this study, FA treatment reduced the TPC content (Figure 4A) and downregulated the expressions of *C4H, 4CL*, and *CHS* genes (Figures 3D–L). The results suggest that FA treatment might reduce the phenolic biosynthesis by suppressing the expressions of *C4H, 4CL*, and *CHS*. The decreased phenolics may restrict the phenolic oxidation reactions, which contribute to the browning alleviation in fresh-cut taros (24). Whether FA can regulate the activity of those enzymes at the protein level remains to be studied.

The discoloration of most agricultural products is mainly attributed to the oxidation of phenolic compounds by PPO (49–51). Postharvest treatments could reduce and/or inhibit PPO activity, which can restrain the browning of fresh-cut fruit or vegetables because PPO is a key enzyme responsible for the formation of dark-colored quinones in plants (52). For example, maclurin treatment suppressed the enzymatic browning and PPO activity in potato supernatant by directly binding to the active site of PPO by forming multiple hydrogen bonds and aromatic interactions with the binding pocket (49). Melatonin treatment reduces PPO activity and gene expressions in fresh-cut pears (53) and taros (4). In addition, the application of 10 mM FA significantly inhibits PPO activity and the yellowing of fresh-cut CWCs (31). In this study, 10 mM FA treatment reduced SQ content and PPO activity and reduced the expression levels of three *PPO* genes in taro slices during cold storage as well (Figures 4B–F). These results suggest that FA treatment might reduce the oxidation of phenolic compounds by suppressing PPO activity, leading to a delay in taro tissue browning.

Many studies have demonstrated that FA can act as a feedback inhibitor of enzymes in the phenolic biosynthesis and oxidation reactions (47). In this study, at concentrations lower and higher than 10 mM, FA treatments did not significantly reduce the surface browning of fresh-cut taros (Figure 1). These results imply that low concentrations of FA might not be sufficient for the inhibition of enzyme activity in the phenolic biosynthesis pathway. On the contrary, FA could act as a substrate of oxidative enzymes such as PPO to form dark-colored pigments when the FA concentrations were higher than a specific concentration such as 10 mM, thus resulting in aggravated browning. Therefore, only proper FA concentration could effectively suppress the browning of fresh-cut taros.

Malondialdehyde content is an indicator of the cell membrane lipid peroxidation (54). LOX is involved in the regulation of lipid peroxidation in fresh-cut fruits and vegetables (55), and the enhanced LOX activity is found to be related to the aggravated browning in fresh-cut foods as seen in lotus roots (56), pear fruits (57), guava fruits (58), and taros (24). LOX catalyzes the first oxygenation step of polyunsaturated fatty acids and results in the formation of hydroperoxides (59, 60). In this study, FA treatment decreased MDA contents, LOX activity, and the expression levels of four *LOX* genes in fresh-cut taros (Figure 6). These results suggest that FA treatment could reduce the membrane lipid peroxidation in fresh-cut taros, thereby maintaining the integrity of the cell membrane and the compartmentation of enzymes and substrates.

Reactive oxygen species (ROS) overproduction or scavenger system failure is one of the main reasons for causing enzymatic browning in fresh-cut products (61). CAT and POD are the two main antioxidant enzymes and play crucial roles in scavenging ROS in plant cells (37, 62). The enhancement of the activities of various antioxidant enzymes such as POD, CAT, and APX could reduce ROS levels and prevent oxidative damage caused by ROS (63). In this study, 10 mM FA treatment significantly enhanced POD and CAT activities and gene expressions and reduced H_2_O_2_ contents in fresh-cut taros (Figure 5). These results show that FA treatment activated the antioxidant system in taro slices, reducing oxidative damages caused by cutting and peeling, which greatly contributed to the reduced browning. The improved antioxidant activity by FA treatment might also contribute to the reduction of membrane lipid peroxidation in fresh-cut taros due to the strong oxidation activity of ROS (64).

Besides ROS scavenging activity, POD is also involved in the oxidation of phenolics (65, 66). However, the available results regarding the roles of POD in the browning of fresh-cut foods were inconsistent. Hydrogen sulfide treatment inhibited POD activity and thus retarded the browning of fresh-cut lotus root slices (35) and CWCs (61) during cold storage. The reduction of carambola fruit browning under UV-C treatment was not due to a reduction in PAL and/or POD activities (67). The cinnamic acid treatment enhances POD activity and gene expression but reduces the browning of fresh-cut taros (22). POD activities and gene expression of fresh-cut taros were activated by 10 mM FA treatment in this study (Figures 5F–H). These results suggest that POD is more suitable to act as an ROS scavenger rather than a browning enhancer in fresh-cut taros. The results reported by Gao et al. concurred with our results and showed that 24-epibrassinolide treatment reduced the surface browning of fresh-cut lotus roots by enhancing POD activity (56).

Taro is characterized by a distinctive aroma after cooking (68), so the aroma is an important characteristic of the high quality of taro. Aromatic compounds are closely related to ethylene production in postharvest fruit (69), and cutting and peeling operations can induce the production of endogenous ethylene, but cold storage shows effective inhibition of ethylene generation in fresh-cut products (70). Therefore, it seems to be unclear how aromatic compounds change in fresh-cut foods under cold storage conditions. In this study, we found that the aromatic quality of DW-treated taros was reduced following a long period of cold storage (Supplementary Table 2 and Figure 7). However, FA treatment prevented the decrease in the number and content of aromatic components. Furthermore, two novel aromatic compounds, namely, non-anal and octanoic acid ethyl ester, were identified in the FA-treated taros (Supplementary Table 2 and Figure 7). Octanoic acid ethyl ester is an important aroma-producing substance in strawberry fruit (71). These results suggest that FA treatment improved the aroma quality of cold-stored fresh-cut taros. LOX is also involved in the formation of some aromatic components (72). In this study, LOX activity was enhanced in FA treatment within the first 3 days (Figure 6B), implying that the enhanced LOX activity at the initial phase of cold storage might be involved in the formation of aroma components in fresh-cut taros. However, how FA treatment influences aromatic components in cold-stored taros remains to be elucidated in the future.

## Conclusion

Treatment with 10 mM FA significantly maintained the L^*^ values but reduced a^*^ values, b^*^ values, and BI in fresh-cut taro slices, suggesting that FA treatment restrained the browning of fresh-cut taro. FA treatment induced the PAL activity but reduced the phenolic biosynthesis by suppressing the expression of *C4H, 4CL*, and *CHS* genes. FA treatment reduced PPO activity and thus reduced the formation of dark-colored quinones in taros. FA treatment reduced oxidative damages caused by cutting by enhancing CAT and POD activities and reducing LOX activity. Moreover, the application of FA improved the aroma quality of cold-stored taro slices. Overall, our results highlight that the FA treatment at proper concentration is probably a useful strategy to maintain the quality of fresh-cut taro and inhibit browning, which is still a big problem in fresh-cut foods. Whether and how FA treatment enhances the nutritional quality of fresh-cut taros should be studied in the future.

## Data Availability Statement

The original contributions presented in the study are included in the article/Supplementary Material, further inquiries can be directed to the corresponding authors.

## Author Contributions

BW: conceptualization, investigation, formal analysis, writing—original draft, review, and editing. YH, ZZ, and JX: investigation. YX: supervision and writing—review and editing. All authors contributed to the article and approved the submitted version.

## Conflict of Interest

The authors declare that the research was conducted in the absence of any commercial or financial relationships that could be construed as a potential conflict of interest.

## Publisher's Note

All claims expressed in this article are solely those of the authors and do not necessarily represent those of their affiliated organizations, or those of the publisher, the editors and the reviewers. Any product that may be evaluated in this article, or claim that may be made by its manufacturer, is not guaranteed or endorsed by the publisher.
