# Supplementary material for: Ferulic Acid Treatment Maintains the Quality of Fresh-Cut Taro (Colocasia esculenta) During Cold Storage
Source: Front Nutr. 2022 May 24;9:884844. doi: 10.3389/fnut.2022.884844 (PMC9172584; doi:10.3389/fnut.2022.884844)
Supplement: Supplementary file 1 [file Table_1.DOC]

Table S1 Primer sequences used in this study.

| Genes | Forward primer sequence (5'-3') | Reverse primer sequence (5'-3') | Product size (bp) |
| --- | --- | --- | --- |
| *CeCAT1* | ACCACAGTCATGCCACTCAG | TCAGGCCATGTCTTCGTGAC | 145 |
| *CeCAT2* | ATTTCCCTTCACGGCACGAT | ACCTTCTGCCCAAGAGAAGC | 109 |
| *CePOD1* | GGGTGCACAGGATGAGTCAA | TAAACACGTCCGCGAAGTCA | 87 |
| *CePOD4* | GCTTCTACTCGAGCTCCTGC | TGGACGCATCACAACCTTGA | 144 |
| *CePOD-N* | GTCGACGACTTCGTATCCCC | TGACTGCTTCGTCAATGGCT | 109 |
| *CePAL1* | GAGGGTCTCGCAATGGTGAA | CTGCATGACCTCGCAGAAGA | 126 |
| *CePAL like* | CATGCGGATGACCTCCAACT | GAAGCAGGATAGGTACGCCC | 70 |
| *CePPO1* | ATCTCCTGGAGCAGCTCTGA | GCAATGTGCCCCACAAGAAG | 94 |
| *CePPO3* | GTGTCCCAGTTCCAGAAGGG | TTCCACCGGTGCTACCTCTA | 92 |
| *CePPO2* | GTCCTACCAACTGCTGTCCC | AGGTACTCGGAGTCCACCAG | 112 |
| *CeC4H1* | TGGTCCCACACATGAACCTG | GTTGACGACCACCTTGGACT | 77 |
| *CeC4H2* | CGATGGTGGAGTGCTTCAGT | CCCAATCCTGGGCATCATCA | 134 |
| *CeC4H3* | CAGGACGAAGGCCTTGATGT | TTGCTGCTTGACATAGCCGA | 75 |
| *Ce4CL1* | TGGGTCTCGGCATAGGTGTA | TCCACGCTTACTGCTTCGAG | 97 |
| *Ce4CL2* | TCGATCGAGCAGTCAGCATC | CCCCAGCTGGTCCATTTGAT | 84 |
| *Ce4CL3* | CAAGGGGGTGATGGTGACTC | CACAGCGTTCAGCGAGTAGA | 145 |
| *CeCHS1* | AAGAGGGAGTTCCAGTCGGA | GGTAGGCCTCACCTTTCACC | 117 |
| *CeCHS2* | GAGCCGACTACCAGCTCATC | CAGCCCTGGTGGTACAACAT | 76 |
| *CeCHS3* | CATGATCGAGGAGATCCGCC | TGGTACAAGACGTTGGACGG | 93 |
| *CeLOX1* | GAACCTTACGTTGGCCAGGA | AAGAACCAACAGGCCACCAA | 73 |
| *CeLOX3* | TGCAACAATGGAGCCGTTTG | GTGTGGGCTCAGTAGCTTGT | 82 |
| *CeLOX4* | CTTCCTCAAGGCTGATGGCA | GGTATACACCTCGCTGACGG | 100 |
| *CeLOX5* | CTCCGAGTACAGGTCCTCCA | TCATCAAAGTCTCGGCCCAC | 122 |
| *CeActin7* | GTCCTACCAACTGCTGTCCC | AGGTACTCGGAGTCCACCAG | 112 |
