# Supplementary material for: Ferulic Acid Treatment Maintains the Quality of Fresh-Cut Taro (Colocasia esculenta) During Cold Storage
Source: Front Nutr. 2022 May 24;9:884844. doi: 10.3389/fnut.2022.884844 (PMC9172584; doi:10.3389/fnut.2022.884844)
Supplement: Supplementary file 2 [file Table_2.DOC]

Table S2 The main volatiles identified by GC-MS analyzer in fresh-cut taros of control (DW) and FA treatment

| No. | Compounds | Retention time (min) | CAS number | Relative contents (%)* | | |
| --- | --- | --- | --- | --- | --- | --- |
|  |  |  |  | 0 d | 12 d | |
|  |  |  |  |  | DW | FA |
| 1 | 2,4-Diamino-N,N,5-trimethyl-6-quinolinesulfonamide | 7.883 | 092144-19-9 | 37.86 | - | - |
| 2 | 1-(2-Acetoxyethyl)-1-(4-methylpent-4-enyl)-2-(1-methylethenyl)cyclobutane | 8.631 | 1000383-49-3 | 18.74 | - | - |
| 3 | Nonanal | 10.748 | 000124-19-6 | - | - | 23.86 |
| 4 | Cyclopentasiloxane, decamethyl- | 12.121 | 000541-02-6 | 11.19 | - | 13.30 |
| 5 | Octanoic acid, ethyl ester | 13.316 | 000106-32-1 | - | - | 41.63 |
| 6 | 2-Benzo[1,3]dioxol-5-yl-8-methoxy-3-nitro-2H-chromene | 21.121 | 1000275-63-1 | 7.80 | - | - |
| 7 | 2,4-Di-tert-butylphenol | 21.484 | 000096-76-4 | 13.61 | - | 10.79 |
| 8 | 2H-Indeno[1,2-b]furan-2-one, 3,3a,4,5,6,7,8,8b-octahydro-8,8-dimethyl | 21.49 | 1000196-74-3 | - | 49.21 | - |
| 9 | 18-Methyl-nonadecane-1,2-dio, trimethylsilyl ether | 25.013 | 1000336-75-9 | 10.79 | 50.79 | 10.42 |

“-” means this compound was not detected in the corresponding treatment.

*The percentage in total identified compound in the corresponding treatment.
